# Prevalence of depressive and anxiety symptoms in chronically ill children and adolescents during COVID-19 pandemic and lockdown: Preliminary findings of a pediatric hospital in Italy

**DOI:** 10.1192/j.eurpsy.2021.731

**Published:** 2021-08-13

**Authors:** C. Correale, M. Di Pastena, I. Tondo, F. Milo, F. Santato, G. Amodeo, T. Grimaldi Capitello, F. Vigevano, S. Cappelletti

**Affiliations:** 1 Clinical Psychology, Bambino Gesù Children Hospital, Rome, Italy; 2 Neurological Sciences, Bambino Gesù Children Hospital, Rome, Italy

**Keywords:** COVID-19, Anxiety, Depression, pediatric chronic illness

## Abstract

**Introduction:**

Psychological problems are common among the pediatric population suffering from a chronic illness, especially compared to the normal population. Stressful life events, such as social distancing measures implemented to counter the COVID-19 pandemic emergency, can strongly influence their epidemiology.

**Objectives:**

The aim of this study was to assess the prevalence rate of depressive and anxiety symptoms among an Italian pediatric population affected by chronic illness and already under a Children Hospital psychological follow-up program during COVID-19 lockdown.

**Methods:**

We conducted a cross-sectional study among 54 Italian children and adolescents affected by chronic illness (mean age: 15y; range: 8.9-18y) during the COVID-19 epidemic period. We assessed depressive and anxiety symptoms with the Patient Health Questionnaire (PHQ-9) and the Generalized Anxiety Disorders (GAD-7) questionnaire during scheduled follow-up checks or teleconsulting.

**Results:**

Preliminary results showed an elevated prevalence of depressive and anxiety symptoms (51% and 48% respectively) among chronically ill children during the COVID-19 outbreak. When compared with a non-ill pediatric population (Zhou et al. 2020), rates are + 7.3% higher for depression and + 10.6% for anxiety.

**Conclusions:**

Chronic ill pediatric patients are a vulnerable group and require careful consideration. For this reason, the healthcare system should be able to implement and guarantee adequate mental health support programs and continuity of care. Further research is necessary since the COVID-19 outbreak could be repeated.

